# Prevalence of non-O157:H7 shiga toxin-producing Escherichia coli in diarrheal stool samples from Nebraska.

**DOI:** 10.3201/eid0605.000513

**Published:** 2000

**Authors:** P D Fey, R S Wickert, M E Rupp, T J Safranek, S H Hinrichs

**Affiliations:** University of Nebraska Medical Center, Omaha, Nebraska 68198-5400, USA.

## Abstract

We determined the prevalence of Shiga toxin-producing Escherichia coli (STEC) in diarrheal stool samples from Nebraska by three methods: cefixime-tellurite sorbitol MacConkey (CT- SMAC) culture, enterohemorrhagic E. coli (EHEC) enzyme immunoassay, and stx1,2 polymerase chain reaction (PCR). Fourteen (4.2%) of 335 specimens were positive by at least one method (CT-SMAC culture [6 of 14], EHEC enzyme immunoassay [13 of 14], stx1,2 PCR [14 of 14]). Six contained serogroup O157, while non-O157 were as prevalent as O157 serogroups.


### Prevalence of Non-O157:H7 Shiga Toxin-Producing *Escherichia coli* in Diarrheal Stool Samples from Nebraska

**Paul D. Fey,* R.S. Wickert,* M.E. Rupp, T.J. Safranek,† and S.H. Hinrichs***
*University of Nebraska Medical Center, Omaha, Nebraska, USA; †Department of Health and Human Services, Lincoln, Nebraska, USA

We determined the prevalence of Shiga toxin-producing *Escherichia coli* (STEC) in diarrheal stool samples from Nebraska by three methods: cefixime-tellurite sorbitol MacConkey (CT-SMAC) culture, enterohemorrhagic *E. coli* (EHEC) enzyme immunoassay, and $stx_{1,2}$ polymerase chain reaction (PCR). Fourteen (4.2%) of 335 specimens were positive by at least one method (CT-SMAC culture [6 of 14], EHEC enzyme immunoassay [13 of 14], $stx_{1,2}$ PCR [14 of 14]). Six contained serogroup 0157, while non-0157 were as prevalent as 0157 serogroups.

Disease caused by Shiga toxin-producing *Escherichia coli* (STEC) ranges from self-limiting diarrhea to hemorrhagic colitis and hemolytic uremic syndrome (HUS). Serotype O157:H7, the most frequently implicated STEC causing hemorrhagic colitis and HUS, has been isolated from large foodborne outbreaks, as well as sporadic cases, in North America and abroad. However, 60 STEC serotypes have been implicated in diarrheal disease, and several non-O157:H7 serotypes have been implicated as the cause of foodborne outbreaks and HUS in the United States, Europe, and Australia. Studies from Canada, Europe, Argentina, and Australia suggest that non-O157:H7 STEC infections are as prevalent, or more so, than O157:H7 infections.

*E. coli* O157:H7 is easily differentiated from other *E. coli* by its inability to rapidly ferment sorbitol; however, non-O157:H7 STEC are phenotypically similar to commensal nonpathogenic *E. coli* and are not detected with sorbitol MacConkey agar. To detect non-O157:H7 STEC, nonculture methods are used (enzyme immunoassay [EIA] or polymerase chain reaction [PCR]), which are typically only performed in reference laboratories. The purpose of this study was to determine the prevalence of non-O157:H7 STEC in persons with diarrhea in Nebraska.

Address for correspondence: Paul D. Fey, University of Nebraska Medical Center, Nebraska Public Health Laboratory, 985400 Nebraska Medical Center, Omaha, NE 68198-5400, USA; fax: 402-559-5581; e-mail: pfey@unmc.edu.

### The Study

Nine regional clinical microbiology laboratories in Nebraska sent stool samples from March 1, 1998, to October 31, 1998, to the Nebraska Public Health Laboratory, University of Nebraska Medical Center. All stool samples that were sent to a participating laboratory with a physician's order to screen for enteric pathogens were included. Thus, all samples were from patients with a differential diagnosis of bacterial gastroenteritis. Patients who had been in the hospital for >2 days before diarrhea developed were excluded. The samples were added to a Para-Pak C&S stool transport container (Meridian Diagnostics, Cincinnati, OH) and sent by courier to the Nebraska Public Health Laboratory.

Samples were plated to cefixime-tellurite sorbitol MacConkey (CT-SMAC) agar plates and screened for typical sorbitol-negative colonies (1). Presumptive colonies were identified as *E. coli* by API strips (Biomerieux Vitek, Hazelwood, MO) and serotyped with RIM *E. coli* O157:H7 (Remel, Lenexa, KS). Samples were injected into 10 mL MacConkey broth (Difco, Detroit, MI) and incubated overnight at 37°C. The Premier enterohemorrhagic *E. coli* (EHEC) assay was performed by using 50 µL of overnight growth. The reaction mixtures were read spectrophotometrically at 450 nm and scored as positive or negative. PCR was performed by first extracting DNA from the overnight culture of MacConkey broth using a QIAamp Tissue kit (Qiagen, Santa

Clarita, CA). The following set of primers, which detects both $stx_1$ and $stx_2$, was used: 5'TTTACG ATAGACTTCTCGAC3' and 5'CACATATAA TTA TTTCGCTC3' (2). *E. coli* O157:H7 strain G5244 was used as positive control (Centers for Disease Control and Prevention [CDC] strain collection). Samples Shiga-toxin positive by either EHEC enzyme EIA, PCR, or both were plated onto sheep-blood agar (Remel) and streaked for isolation. After overnight growth, multiple *E. coli*-like colonies were selected for retesting by using the Premier EHEC assay. Positive colonies were identified to species level by using API strips and serotyped by CDC.

Multiplex PCR (3) was performed on isolated Shiga toxin-positive colonies to detect specific genes encoding Shiga toxins 1 and 2 ($stx_1$ and $stx_2$), intimin (*eaeA*), and enterohemolysin A (*ehxA*). Genomic DNA suitable for pulsed-field gel electrophoresis (PFGE) was prepared (4) and digested with *Xba*I (Roche, Indianapolis, IN). *E. coli* O157:H7 G5244 was used as a standard. PFGE patterns were captured by a Bio-Rad Gel-Doc system and were analyzed by Molecular Analyst software (Bio-Rad, Hercules, CA).

Of the nine clinical laboratories that submitted 335 samples during the study period, five submitted samples positive by CT-SMAC culture, EIA, or *stx* PCR (Table 1). Fourteen 4.2%) samples were positive by at least one of the methods; 13 of these were obtained either through direct culture by using CT-SMAC or through Shiga toxin screening and subsequent colony isolation. Six of the thirteen were serotype O157:H7 or O157:NM; seven were non-O157 serotypes. All seven of the non-O157 isolates were the predominant species found in the culture when the sample was plated on sheep-blood agar. All six *E. coli* O157:H7 or O157:NM isolates were detected by using CT-SMAC culture and *stx* PCR; five of six were detected by EIA. All of seven non-O157 isolates were detected by EIA or *stx* PCR. One sample (isolate B3) that was negative by EIA but positive by PCR and CT-SMAC culture was subsequently found to be positive by EIA when tested individually. One sample from laboratory C was positive for EIA and *stx* PCR (both tests were weak positives), but no Shiga toxin-positive colony was obtained upon repeat subculture. The low prevalence of this organism in the stool sample may reflect STEC carriage in this patient. By the combined results of both culture and EIA as the reference standard (14 samples positive, 321 samples negative), the sensitivity and specificity of the *stx* PCR (14 samples positive, 321 samples negative) were each 100%.

PCR was performed on the 13 isolated STEC to detect $stx_1$, $stx_2$, *eae,* and *ehxA* (Table 2). All isolates, regardless of serotype, encoded *eae* and *ehxA*; three of five O157 isolates encoded both $stx_1$ and $stx_2$; two of seven non-O157:H7 isolates encoded $stx_2$ (both O111:NM). PFGE showed that all but two STEC isolates were unrelated; isolates D1 and D2 (both O111:NM), which were isolated from samples sent from the same laboratory, were indistinguishable (data not shown).

Table 1. Results of CT-SMAC culture, enzyme immunoassay, and *stx* polymerase chain reaction

| Laboratory | Isolate | Serotype | CT-SMAC[a] | EIA | PCR |
|---|---|---|---|---|---|
| A | A1 | O157:H7 | + | + | + |
| | A2 | O157:H7 | + | + | + |
| | A3 | O26:H11 | - | + | + |
| | A4 | O157:NM | + | + | + |
| B | B1 | O145:NM | - | + | + |
| | B2 | O103:H2 | - | + | + |
| | B3 | O157:NM | + | - | + |
| C | NI[b] | NI | - | + | + |
| D | D1 | O111:NM | - | + | + |
| | D2 | O111:NM | - | + | + |
| | D3 | O157:H7 | + | + | + |
| | D4 | O157:H7 | + | + | + |
| E | E1 | Orough:H2 | - | + | + |
| | E2 | O26:H11 | - | + | + |

[a](+) Denotes a positive CT-SMAC culture, EIA, or *stx* PCR; (-) denotes a negative CT-SMAC culture, EIA, or *stx* PCR; CT-SMAC = cefixime-tellurite sorbitol MacConkey agar; EIA = enzyme immunoassay; PCR = polymerase chain reaction.
[b]NI = Not isolated.

Table 2. Results from multiplex polymerase chain reaction (PCR) amplification

| Isolate | Serotype | *stx1*[a] | stx2 | *eae* | *ehxA* |
|---|---|---|---|---|---|
| A1 | O157:H7 | + | + | + | + |
| A2 | O157:H7 | - | + | + | + |
| A3 | O26:H11 | + | - | + | + |
| A4 | O157:NM | + | + | + | + |
| B1 | O145:NM | + | - | + | + |
| B2 | O103:H2 | + | - | + | + |
| D1 | O111:NM | + | + | + | + |
| D2 | O111:NM | + | + | + | + |
| D3 | O157:H7 | - | + | + | + |
| D4 | O157:H7 | + | + | + | + |
| E1 | Orough:H2 | + | - | + | + |
| E2 | O26:H11 | + | - | + | + |

[a](+) Denotes presence of gene as assessed by PCR; (-) denotes absence of gene as assessed by PCR.

### Conclusions

In a 1997 study of 30,000 diarrheal stool samples, *E. coli* O157:H7 was the fourth most prevalent bacterial enteric pathogen (5). However, the incidence of non-O157 STEC in the United States is not well established. Studies from Europe have shown that the prevalence of STEC in diarrheal samples is 0.3% to 9.3%; serogroup O157 prevalence is 0% to 2.7%. In Australia, serotype O111:NM is the most frequent cause of serious human disease and has been associated with outbreaks. In a recent study of 3,289 diarrheal samples from clinical laboratories in the United States, non-O157 STEC were more prevalent than O157 serotypes (6). STEC was found to be as prevalent (1.2%) as *Shigella* sp. (1.4%), and almost as prevalent as *Salmonella* sp. (2.4%) and *Campylobacter* sp. (2.0%). Testing for *E. coli* O157:H7 alone would have missed up to 50% of STEC.

Our study is the first to address the prevalence of non-O157 STEC in diarrheal samples from the Great Plains region of the northern United States, where cattle and other animal reservoirs of STEC are abundant. In our study, 4.2% of the samples were positive for STEC by CT-SMAC culture, PCR, or Meridian EHEC EIA. Though this prevalence is higher than previously reported in the United States, other studies have shown that northern states have a higher prevalence of *E. coli* O157:H7 (7). In addition, Nebraska has a large rural population, whose members may have contact with animal reservoirs that carry STEC. Five different non-O157 STEC serotypes were isolated: O111:NM, O26:H11, O145:NM, O103:H2, and Orough:H2. Four of these are associated with HUS (O111:NM [8–10], O26:H11[11], O145:NM [12], and O103:H2 [13]). In addition, serotypes O111:NM and O26:H11 have been associated with diarrhea in weaned calves (14). Although most STEC cases are linked to eating undercooked hamburger (15), contact with food animals has also been implicated as a source of infection (16).

Shiga toxins 1 and 2 are the main virulence factors associated with hemorrhagic colitis and HUS, presumably because they interact with endothelial cells at the site of infection and in the glomeruli and arterioles of the kidney (17). $stx_1$ and $stx_2$ are highly related yet immunologically distinct. STEC produce other accessory virulence factors, including intimin (*eae*) and enterohemolysin A (*ehxA*). The former is responsible for the characteristic histopathologic feature known as the attaching and effacing (A/E) lesion (18); *ehxA* is a hemolysin encoded by the 90-kb virulence-associated plasmid found in most STEC infecting humans (19). A study of 237 isolates from 118 serotypes found a significant association between $stx_2$ and *eae* in isolates that caused hemorrhagic colitis and HUS in humans (20) and between *ehxA* and severe disease. STEC isolates from asymptomatic human carriers usually do not encode *eae* and therefore may not have a mechanism to adhere to intestinal epithelial cells (21). However, STEC isolates lacking the *eae* gene have been associated with hemorrhagic colitis and HUS (22). All STEC isolates obtained in this study encoded both *eae* and *ehxA*. The six O157 isolates also encoded $stx_2$, as did both O111:NM isolates, which indicates that at least these eight isolates could produce serious disease. Five of seven non-O157 isolates encoded $stx_1$ only; however, STEC isolates associated with hemorrhagic colitis and HUS that encoded only $stx_1$ have been reported (23).

All O157:H7, O157:NM, and O26:H11 isolates in our study were distinct by PFGE RFLP patterns, which suggests that these cases were sporadic. However, two O111:NM isolates (D1 and D2) from the same community had indistinguishable PFGE RFLP patterns. The isolates were from different patients and were cultured within 1 day of each other. This suggests that an outbreak of O111:NM may have occurred that was not detected by standard laboratory techniques. However, without epidemiologic information, these results are difficult to interpret.

This study demonstrated that non-O157 STEC serotypes are at least as prevalent as serogroup O157 in diarrheal samples from Nebraska. Non-O157 STEC isolates presumably were the cause of diarrhea in 7 of 14 positive samples. These non-O157 isolates carried known STEC virulence genes and were the predominant organism found in culture. Other bacterial pathogens such as *Salmonella*, *Shigella*, and *Campylobacter* were not isolated from these seven samples. Our results suggest that *stx* PCR is as sensitive and specific as CT-SMAC culture and EIA combined, and therefore may be used as an alternate method to diagnose diarrheal infections caused by STEC. Clinical laboratories may need to implement serotype-independent methods to avoid underdiagnosis of STEC-mediated bacterial gastroenteritis.

Dr. Fey is an assistant professor in infectious disease and pathology and microbiology and the associate director of the Nebraska Public Health Laboratory at the University of Nebraska Medical Center. His interests include the epidemiology and antibiotic resistance of diarrheal pathogens as well as the genetics and pathogenesis of staphylococci.

### References

1. Zadik PM, Chapman PA, Siddons CA. Use of tellurite for the selection of verocytotoxigenic *Escherichia coli* O157. J Med Microbiol 1993;39:155-8.
2. Fratamico PM, Sackitey SK, Wiedmann M, Deng MY. Detection of *Escherichia coli* O157:H7 by multiplex PCR. J Clin Microbiol 1995;33:2188-91.
3. Fagan PK, Hornitzky MA, Bettelheim KA, Djordjevic SP. Detection of Shiga-like toxin (*stx*$_1$ and *stx*$_2$), intimin (*eaeA*), and enterohemorrhagic *Escherichia coli* (EHEC) hemolysin (EHEC *hlyA*) genes in animal feces by multiplex PCR. Appl Environ Microbiol 1999;65:868-72.
4. Gautom RK. Rapid pulsed-field gel electrophoresis protocol for typing of *Escherichia coli* O157:H7 and other gram-negative organisms in 1 day. J Clin Microbiol 1997;35:2977-80.
5. Slutsker L, Ries AA, Greene KD, Wells JG, Hutwagner L, Griffin PM, et al. *Escherichia coli* O157:H7 diarrhea in the US: clinical and epidemiological features. Ann Intern Med 1997;126:505-13.
6. Acheson DW, Frankson K, Willis D, STEC Prevalence Study Group. Multicenter prevalence study of Shiga toxin-producing *Escherichia coli.* Abstracts of the 98th General Meeting for the American Society for Microbiology. Washington: American Society for Microbiology;1998. [Abstract C-205]
7. Griffin PM. *Escherichia coli* O157:H7 and other enterohemorrhagic *Escherichia coli.* In: Blaser MJ, Smith PD, Raudin JI, Greenburg HB, Guerrant RL, editors. Infections of the gastrointestinal tract. New York: Raven Press;1995. p. 739-61.
8. Boudaillez B, Berquin P, Mariani-Kurkdjian P, Bef D, Cuvelier B, Capek I, et al. Possible person to person transmission of *Escherichia coli* O111-associated hemolytic uremic syndrome. Pediatr Nephrol 1996;11:36-9.
9. Caprioli A, Luzzi I, Rosmini F, Resti C, Edefonti A, Perfumo F, et al. Community-wide outbreak of hemolytic-uremic syndrome associated with non-O157 verotoxin-producing *Escherichia coli.* J Infect Dis 1994;169:208-11.
10. Robbins-Browne RM, Elliott E, Desmarchelier P. Shiga toxin-producing *Escherichia coli* in Australia. In: Kapar JP, O'Brien AD, editors. *Escherichia coli* O157:H7 and other Shiga toxin-producing *E. coli* strains. Washington: American Society for Microbiology;1998. p.66-72.
11. Sramkova LM, Bielaszewska M, Janda J, Blahova K, Hausner O. Vero cyctotoxin-producing strains of *Escherichia coli* in children with hemolytic uremic syndrome and diarrhoea in Czechoslovakia. Infection 1990;18:204-9.
12. Karmali MA, Petric M, Lim C, Fleming PC, Arus GS, Lior H. The association between idiopathic hemolytic uremic syndrome and infection by verotoxin-producing *Escherichia coli.* J Infect Dis 1985;151:775-82.
13. Tarr PI, Fouser LS, Stapleton AE, Wilson RA, Kim HH, Vary JC Jr, et al. Hemolytic uremic syndrome in a six-year-old girl after a urinary tract infection with Shiga toxin-producing *Escherichia coli* O103:H2. N Engl J Med 1996;335:635-8.
14. Kudva IT, Hatfield PG, Hovde CJ. Characterization of *Escherichia coli* O157:H7 and other Shiga toxin-producing *Escherichia coli* serotypes isolated from sheep. J Clin Microbiol 1997;35:892-9.
15. Slutsker L, Ries AA, Maloney K, Wells JG, Greene KD, Griffin PM, et al. A nationwide case-control study of *E. coli* O157:H7 infection in the United States. J Infect Dis 1998;177:962-6.
16. Trevena WB, Willshaw GA, Cheasty T, Wray C. Associations between human infection with vero cytotoxin-producing *Escherichia coli* O157:H7 and farm animal contact. In: Proceedings of the 3rd international symposium and workshop on Shiga-toxin (verocytotoxin)-producing *Escherichia coli* infections. 1997. abstract V28/I.
17. Kaplan BS, Cleary TG, Obrig TG. Recent advances in understanding the pathogenesis of the hemolytic uremic syndrome. Pediatr Nephrol 1990;4:276-83.
18. McDaniel TK, Kaper JB. A cloned pathogenicity island from enteropathogenic *Escherichia coli* confers the attaching and effacing phenotype of *E. coli* K-12. Mol Microbiol 1997;23:399-407.
19. Boerlin P, Chen S, Colbourne JK, Johnson R, De Grandis S, Gyles C. Evolution of enterohemorrhagic *Escherichia coli* hemolysin plasmids and the locus for enterocyte effacement in Shiga toxin-producing *Escherichia coli.* Infect Immun 1998;66:2553-61.
20. Boerlin P, McEwen SA, Boerlin-Petzold F, Wilson JB, Johnson RP, Gyles CL. Associations between virulence factors of Shiga toxin-producing *Escherichia coli* and disease in humans. J Clin Microbiol 1999;37:497-503.
21. Stephen R, Untermann F. Virulence factors and phenotypical traits of verotoxin-producing *Escherichia coli* strains isolated from asymptomatic human carriers. J Clin Microbiol 1999;37:1570-2.
22. Paton AW, Woodrow MC, Doyle RW, Lanser JA, Paton JC. Molecular characterization of Shiga toxigenic *Escherichia coli* O113:H21 strain lacking *eae* responsible for a cluster of cases of HUS. J Clin Microbiol 1999;37:3357-61.
23. Beutin L, Zimmermann S, Gleier K. Human infections with Shiga toxin-producing *Escherichia coli* other than serogroup O157 in Germany. Emerg Infect Dis 1998;4:635-9.
